# Supplementary material for: Scoping review protocol to map evidence on South-South learning exchange in family planning
Source: BMJ Open. 2023 Mar 23;13(3):e061685. doi: 10.1136/bmjopen-2022-061685 (PMC10040009; doi:10.1136/bmjopen-2022-061685)
Supplement: Supplementary data [file bmjopen-2022-061685supp002.pdf]

Interview guide for Stakeholder consultations on SSLE in family planning

May I remind you that:

1) The interview will be audio recorded to ensure all information is captured. I would like to ask for your consent before we proceed.
2) You are free to refuse to participate and may withdraw your consent or discontinue participation at any time. The refusal to participate will not result in any penalty or loss of benefits. You can also skip any questions you do not want to answer.

Name of Participant:

Organization and designation:

Date & time of interview:

<u>Ice breaker</u>

1. Welcome and introductions
2. Describe the role of your organization in the South-South Learning Exchange.
3. Describe your role in the SSLE process and the number of SSLEs you were involved in.

<u>Purpose of SSLE in family planning</u>

4. Explain the main goal of each SSLE process you were involved in.
   Probes: Area: health system service, health commodities, human resource management and development, health care delivery, health care financing, health information system, etc

<u>Process of SSLE in family planning</u>

5. How did the process begin?
   Probes: How were the countries selected? By whom? Who were the knowledge seekers and providers?
6. Why did the process begin?
   Probes: How did the country identify the learning area/ objective of the SSLE? Did the country conduct a needs assessment/situational analysis?
7. When did this SSLE take place, and how long was the duration?
8. List the stakeholders involved in SSLE and their roles.
9. Who were the main champions of the process (NGO, Ministry of Health, etc.)?
10. Did you follow a standardized process for conducting the SSLE?

Probes: Was a structured approach or a Standard Operation Procedure used to guide the SSLE? Describe the approach adopted throughout the process.

11. Describe how the process was monitored and how were the results measured?

    Probes: how were the indicators selected and tracked, and how often?

Key outcomes

12. What were the family planning outcomes achieved from SSLE?

    Probes: Were policies, guidelines, capacity building or quality of services achieved at the end of the SSLE process? Was the planned goal achieved?

Barriers and enablers

13. In your opinion, what were the barriers faced during the SSLE process?
14. What were the facilitators that helped during the SSLE process?

Final question

15. Describe the main lessons learnt from your experience with SSLE?

Allagh KP, *et al. BMJ Open* 2023; 13:e061685. doi: 10.1136/bmjopen-2022-061685
