# Supplementary material for: Scoping review protocol to map evidence on South-South learning exchange in family planning
Source: BMJ Open. 2023 Mar 23;13(3):e061685. doi: 10.1136/bmjopen-2022-061685 (PMC10040009; doi:10.1136/bmjopen-2022-061685)
Supplement: Author's manuscript [file bmjopen-2022-061685.draft_revisions.pdf]

BMJ Open

BMJ Open is committed to open peer review. As part of this commitment we make the peer review history of every article we publish publicly available.

When an article is published we post the peer reviewers' comments and the authors' responses online. We also post the versions of the paper that were used during peer review. These are the versions that the peer review comments apply to.

The versions of the paper that follow are the versions that were submitted during the peer review process. They are not the versions of record or the final published versions. They should not be cited or distributed as the published version of this manuscript.

BMJ Open is an open access journal and the full, final, typeset and author-corrected version of record of the manuscript is available on our site with no access controls, subscription charges or pay-per-view fees (http://bmjopen.bmj.com).

If you have any questions on BMJ Open's open peer review process please email info.bmjopen@bmj.com

A scoping review protocol to map evidence on South-South learning exchange in family planning

Journal:	*BMJ Open*
Manuscript ID	bmjopen-2022-061685
Article Type:	Protocol
Date Submitted by the Author:	02-Feb-2022

Complete List of Authors:	Allagh, Komal Preet; World Health Organization<br>Kiarie, James; World Health Organization, Department of Reproductive Health and Research<br>Triulzi, Isotta; Scuola Superiore Sant'Anna, Institute of Managment<br>Kabra, Rita ; World Health Organization, Reproductive Health and Research

Keywords:	Reproductive medicine < GYNAECOLOGY, Public health < INFECTIOUS DISEASES, Protocols & guidelines < HEALTH SERVICES ADMINISTRATION & MANAGEMENT

SCHOLARONE™
Manuscripts

Title: A scoping review protocol to map evidence on South-South learning exchange in family planning

Authors: Komal Preet Allagh[1], James Kiarie[1], Isotta Triulzi[1], Rita Kabra[1]

Affiliations: [1]Department of Reproductive Health and Research including UNDP/UNFPA/UNICEF/WHO/World Bank Special Programme of Research, Development and Research Training in Human Reproduction, World Health Organization, Avenue Appia 20, 1211 Geneva, Switzerland.

Corresponding author: Rita Kabra

Email: kabrar@who.int

For peer review only

Abstract

Introduction

South-South learning exchange (SSLE) is an interactive learning process where stakeholder teams exchange knowledge and experience to help one, or both to work towards change, by identifying, adopting and/or strengthening implementation of a best practice. SSLE has been conducted between countries to share knowledge on best practices and policies in family planning. To the best of our knowledge, no scoping review has been conducted to synthesize evidence on South-South learning exchange in family planning. In this paper, we outline the protocol to conduct scoping review on South-South learning exchange in family planning.

Methods and analysis

Arksey and O'Malley's scoping review framework with adaptions from Levac et al will be used to guide this scoping review. We will search electronic databases (Medline, Embase, CINAHL, Hinari, ProQuest DB, PUBMED, Web of Science, and WorldCat), grey literature sources and reference lists of included studies. The abstract and title screening, full-text screening and data charting will be conducted by two independent reviewers. The findings will be summarized into a narrative based on thematic analysis. Stakeholder interviews will be conducted to understand their perception and experiences in applying South-South learning exchange in family planning.

Ethics and dissemination

The findings from the study will provide useful insights into effective approaches, barriers, facilitators to conduct South-South Learning exchange in family planning. This knowledge will be of significant public health relevance and will help in designing future learning exchanges between countries in the south to accelerate access to

For peer review only - http://bmjopen.bmj.com/site/about/guidelines.xhtml

quality family planning services. The findings will be disseminated via peer-reviewed journals, conference proceedings, newsletters and workshops.

Keywords: Scoping review, south-south learning exchange, family planning, contraception

Strengths and limitations of this study

- This will be the first scoping review to identify effective approaches, barriers, facilitators to conduct South-South Learning exchange in family planning.
- Stakeholders will be consulted and engaged throughout the review process.
- A comprehensive search strategy is developed with the consultation of a chief librarian to promote a sensitive search.
- The quality of the included studies will not be assessed.

For peer review only

For peer review only - http://bmjopen.bmj.com/site/about/guidelines.xhtml

Background

South-South learning exchange (SSLE) is an interactive learning process where stakeholder teams exchange knowledge and experience to help one, or both teams to work towards change, by identifying, adopting and/or strengthening implementation of a best practice [1]. Learning exchange can take place between two teams at the country level or within countries, between provinces, regions, states, districts or local administrative units. The SSLE can offer value to both teams by supporting bi-directional sharing of knowledge, good practices, supporting scaling-up of good practices and capacity building of participants to advocate for the change process.

Since 2019, World Health Organization (WHO) has been conducting SSLE in family planning under the WHO Family Planning Accelerator project [2] using a standardized 5-step methodology [1] designed to ensure that SSLE is country-driven, focused on outputs/outcomes and involves rigorous monitoring of the process. The WHO FP Accelerator project builds on the WHO FP Umbrella project that supported over 50 countries to update their national FP guidelines to the latest WHO recommendations [3].

WHO describes SSLE as 'an interactive exchange of knowledge and experience between two country teams to help one or both teams to work towards a (desired) change' [1]. At the International Conference on Population and Development (ICPD), Cairo in 1994, one of the recommendations was that more attention should be given to South-South cooperation as an important instrument of development [4]. Since then, several countries have reported using SSLE referred to as South-South cooperation (SSC), to improve family planning outcomes using different approaches like study tours, site visits, training or expert visits. Together with political dialogue, technical and financial cooperation, SSLE has assisted in several knowledge and expertise exchanges through programs, projects and initiatives that

For peer review only

have helped solve specific challenges. However, to date, no document highlights and summarizes the key features of an SSLE in family planning, such as the most effective approach to achieve the FP outcomes, the barriers and facilitators to expect from the learning exchange and how to overcome these, and the objectives of the SSLEs that have already taken place.

To the best of our knowledge, there is no existing published synthesis on South-South learning exchange in family planning that incorporates both the reviewed and non-peer-reviewed literature. This scoping review aims to systematically review published and grey literature on SSLE in family planning and identify gaps in the available knowledge to help guide future SSLEs in family planning and research in the field. The study objectives are to review published and grey literature on SSLE in family planning to identify the (i) purposes, (ii) approaches, (iii) key outcomes, (iv) enablers and barriers.

Methods

This scoping review is based on the framework proposed by Arksey and O'Malley's [5] which has been further developed by Levac et al [6]. The framework has six steps: (1) Identification of the research question, (ii)Identification of relevant studies, (iii)Study selection, (iv) Data charting, (v) Collating, summarizing and reporting the results and (vi) Stakeholder consultation

Stage 1: Identification of the research question

This scoping review seeks to answer the following specific research questions:

i. For what purposes have South-South learning exchanges in family planning been used?

For peer review only - http://bmjopen.bmj.com/site/about/guidelines.xhtml
For peer review only

ii. What approaches and methods are used in conducting South-South learning exchange in family planning between countries?

iii. What are the barriers and facilitators encountered in conducting South-South learning exchange in family planning?

iv. What outcomes have been achieved by South-South learning exchange in family planning?

Stage 2: Identification of relevant studies (Search strategy)

With support from the chief librarian at WHO Geneva, the authors will conduct a systematic search within the following electronic databases: Medline, Embase, CINAHL, Hinari, ProQuest DB, PUBMED, Web of Science, and WorldCat. This study will use a complete search strategy that employs keywords, medical subject headings (MeSH) or subject headings search terms that relate to key review concepts: South-South learning exchange, South-South knowledge exchange, South-South exchange, Peer to peer learning exchange, South-South cooperation, information sharing, information exchange, knowledge sharing, knowledge exchange, learning exchange, Family planning, contraception, reproductive health, as well as Boolean terms "AND" and "OR".

No language restrictions will be imposed. Reference lists of the articles will be used to identify more studies using a snowball approach. We will also conduct a grey literature search using Google Scholar and relevant websites (FP 2030, Partners in population and development, UNFPA, WHO, UNOSCC, S-S Galaxy, World Bank and USAID). Types of grey literature that will be retrieved and used in the review include reports, articles, conference proceedings, PhD and Master's thesis, and case studies.

Stage 3: Study Selection

For peer review only - http://bmjopen.bmj.com/site/about/guidelines.xhtml

This review will include studies that meet the defined eligibility criteria (see table 1). No limits will be placed on study period, language and location of study.

Table 1: Eligibility criteria for the scoping review

Criteria	Inclusion	Exclusion
**Country**	Any country	None
**Date**	Any year	None
**Language**	All languages	None
**Research focus**	Studies that mention objectives, purpose, approaches, process, enablers, barriers, and outcomes of SSLE in family planning.	SSLE on topics other than FP
**Geographical location**	SSLE between countries	SSLE within a country (between institutions, cities & districts)
**Document type**	Scientific report, case study, commentaries, research article, conference proceedings, student thesis, letter to editors and reviews.	Newspaper, power point presentations and magazine articles

Selection process:

We will use a two-part study selection process. First, two independent reviewers (RK and KPA) will screen all titles and abstracts as per inclusion/exclusion criteria. Any article that is considered relevant by either or both reviewers will be included for full-text review. The reviewers (RK and KPA) will then independently assess the full text to determine if the study is included. A third reviewer (JK) will resolve any discrepancies that arise between the two reviewers. Zotero reference manager version 5.0.96.3 [7], a citation and bibliographic software will be used to store and organize all references. The PRISMA flow diagram [8] will be used to report the results of the screening.

Stage 4: Data charting

A data charting form will be prepared in Microsoft Excel to extract study characteristics. KPA and RK will extract all relevant data from the included studies after a thorough reading of the full texts. The data charting form will include 14 categories as listed in table 2. This list will be

For peer review only - http://bmjopen.bmj.com/site/about/guidelines.xhtml

updated when we start reviewing the studies to capture all relevant data to answer the review questions.

Table 2: Data extraction framework

Main category	Description
1. Authors	Name of the authors
2. Title	Title of study
3. Source of publication	Title of the journal or name of publishing organization
4. Year of publication	Year of publication
5. Purpose/objective of SSLE	Describe the stated purpose of the SSLE
6. Year and duration of the SSLE	Indicate the year of SSLE and duration the SSLE lasted
7. Countries participating in the SSLE	List the mentor and mentee countries.
8. Approach used for SSLE	Describe the method used to conduct the learning exchange, example: study tour, virtual, reciprocal exchange, etc.
9. Process of SSLE	Describe the stated process of the SSLE for instance: was a standard method used, planning phase, were stakeholders involved, was an action plan developed, was it implemented, was a follow up done after SSLE was completed.
10. Key stakeholders	Indicate the key FP stakeholders involved in the SSLE in both the countries
11. Reported outputs	Describe the family planning outputs reported in the SSLE (e.g.: capacity building, policy change, etc.)
12. Reported outcomes	Describe the family planning outcomes reported in the SSLE (e.g.: contraceptive prevalence rate, unmet need)
13. Barriers	Describe the factors that inhibit the implementation of the SSLE
14. Facilitators	Describe the factors that supported or enabled the implementation of the SSLE

The data charting form will be pilot tested in a few studies to ensure it is capturing all the information accurately.

Stage 5: Collating, summarizing and reporting the results

A narrative account of the included studies will be prepared to present the literature on SSLE in family planning based on a thematic analysis. Each author will independently review the extracted information to summarize the findings presented across the articles. Relevant themes and sub-themes relating to the study objectives will be developed around the following: (i) Purpose of SSLE in family planning, (ii) methods and process used for SSLE in

For peer review only - http://bmjopen.bmj.com/site/about/guidelines.xhtml

family planning, (iii) FP outputs and outcomes achieved from SSLE in family planning and (iv) Barriers and enablers of SSLE in family planning.

Stage 6: Stakeholder Consultation

Levac et al. suggested that the consultation stage provides opportunities for stakeholder involvement, providing insights beyond what is reported in the literature [4]. The consultations will aim to understand stakeholder perception and experiences in applying SSLE in family planning. Following ethical approval, one reviewer (IT) will identify and invite the authors of the included studies for the interviews. In addition, a snowball approach will be used to identify more experts. We will interview 12-20 individuals. All interviews will be conducted virtually via google meet for an approximate duration of 45-60 minutes and will be recorded using a voice recorder and through taking notes. Participants will be assured of confidentiality and verbal informed consent will be recorded. Each interview will be transcribed verbatim. Relevant themes and sub-themes relating to the study objectives will be identified using RQDA software for qualitative analysis [9].

The interviewer will provide an overview of the scoping review. A semi-structured interview guide is developed to understand the experiences and views of stakeholders on SSLE in family planning. The questionnaire includes a set of open-ended questions (supplementary file) to guide the discussion and will cover the following topics: (i) role of the organization in SSLE and the process used (ii) purpose of SSLE and approaches used (iii) views and experiences on SSLE in FP (iv) perception on challenges and successes observed during the SSLE process and (v) lessons learnt.

Patient and public involvement

No patients are involved. The study is a review of literature, there are no study participants.

For peer review only - http://bmjopen.bmj.com/site/about/guidelines.xhtml

Discussion

The proposed review aims to map evidence on South-South learning exchange in family planning. The extracted data will be presented in a narrative and tabular form to cover all the review objectives. The results of this scoping review will build evidence on effective approaches, barriers, facilitators and key outcomes achieved in SSLE in family planning and may reveal further research areas. Results of the scoping review will be disseminated in a peer-reviewed journal and other ways such as through consultations, newsletters, conferences and workshops.

For peer review only

For peer review only - http://bmjopen.bmj.com/site/about/guidelines.xhtml

Abbreviations

FP- Family planning

PRISMA- Preferred Reporting Items for Systematic Reviews and Meta-Analyses

PPD- Partners in Population and Development

SSLE- South-South learning exchange

SSC- South-South Cooperation

S-S Galaxy- South-South Galaxy

WHO- World Health Organization

UNFPA- United Nations Population Fund

UNOSCC- United Nations Office of South-South Cooperation

USAID- United Nations Agency of International Development

Funding:

This work received funding from the World Health Organization (WHO) through the WHO FP Accelerator Project 2019-2022 supported by the Bill and Melinda Gates Foundation [OPP1203035].

The content is solely the responsibility of the authors and does not necessarily represent the official views of the funders. Any opinion, finding, conclusion or recommendation expressed in this material is that of the authors.

Conflict of Interest:

The authors declare no conflict of interest.

Contributions:

KPA prepared the first draft with significant contributions from JK, IT and RK. All authors reviewed the draft manuscript and approved the final manuscript for publication.

For peer review only - http://bmjopen.bmj.com/site/about/guidelines.xhtml

References:

1. IBP Network and World Health Organization. Family planning Accelerator project: A Step-by-Step Guide to South-South learning exchanges. Geneva. World Health Organization. Retrieved on 27th January 2022 from https://ibpnetwork.org/media_center/folder/47ae9fb7-bab0-41fd-8a7d-fb8fd7d65018
2. World Health Organization. WHO Family planning Accelerator project. February 2020. Retrieved on 22nd March 2021 from https://www.who.int/reproductivehealth/projects/WHO-FP-Accelerator-Project-Brief.pdf?ua=1
3. Kabra R., Ali M., Kiarie J. Design and initial implementation of the WHO FP umbrella project- to strengthen contraceptive services in the sub-Saharan Africa. Reproductive Health. 2017. 14:74, DOI 10.1186/s12978-017-0335-0. Accessed on 24 December 2021 from https://reproductive-health-journal.biomedcentral.com/track/pdf/10.1186/s12978-017-0335-0.pdf
4. Evans I. What do you do, Partner? The Lancet. 1996, 348: 211-212
5. Arksey H, O'Malley L. Scoping studies: towards a methodological framework. International Journal of Social Research Methodology. 2005; 8:19-42.
6. Levac D, Colquhoun H, O'Brien KK. Scoping studies: advancing the methodology. Implementation Science 2010; 5: 69.
7. Zotero. Retrieved on 27th January 2022 from https://www.zotero.org/
8. PRISMA-Transparent reporting of Systematic reviews and meta-analyses. Retrieved on 27th January 2022 from http://prisma-statement.org/prismastatement/flowdiagram.aspx

For peer review only - http://bmjopen.bmj.com/site/about/guidelines.xhtml

9. What is RQDA and what are its features? Retrieved on 27th January 2022 from http://rqda.r-forge.r-project.org/

For peer review only

For peer review only - http://bmjopen.bmj.com/site/about/guidelines.xhtml

Interview guide for Stakeholder consultations on SSLE in family planning

May I remind you that:

1) The interview will be audio-recorded to ensure all information is captured. I would like to ask for your consent before we proceed.
2) You are free to refuse to participate, and you may withdraw your consent or discontinue participation at any time. The refusal to participate will not result in any penalty or loss of benefits. You can also skip any questions you do not want to answer.

Name of Participant:

Organization and designation:

Date & time of interview:

<u>Ice breaker</u>

1. Welcome and introductions
2. Describe the role of your organization in the South-South Learning Exchange?
3. Describe your role in the SSLE process and the number of SSLEs you were involved in?

<u>Purpose of SSLE in family planning</u>

4. Explain the main goal of each SSLE process you were involved in?

   Probes: Area: health system service, health commodities, human resource management and development, health care delivery, health care financing, health information system, etc.

<u>Process of SSLE in family planning</u>

5. How did the process begin?

   Probes: How were the countries selected? By whom? Who were the knowledge seeker and provider?
6. Why did the process begin?

   Probes: How did the country identify the learning area/objective of the SSLE? Did the country conduct a needs assessment/situational analysis?
7. When did this SSLE take place and how long was the duration?
8. List the stakeholders involved in SSLE and their role?
9. Who were the main champions of the process (NGO, Ministry of Health, etc)?
10. Did you follow a standardized process for conducting the SSLE?

For peer review only

Probes: Was a structured approach or a Standard Operation Procedure used to guide the SSLE? Describe the approach adopted throughout the process?

11. Describe how the process was monitored and how were the results measured?

    Probes: How were the indicators selected and tracked, and how often?

Key outcomes

12. What family planning outcomes were achieved from SSLE?

    Probes: Were policies, guidelines, capacity building or quality of services in family planning achieved at the end of the SSLE process? Was the planned goal achieved?

Barriers and enablers

13. In your opinion, what were the barriers faced during the SSLE process?
14. What were the facilitators that helped during the SSLE process?

Final question

15. Describe the main lessons learnt from your experience with SSLE?

For peer review only

For peer review only - http://bmjopen.bmj.com/site/about/guidelines.xhtml

BMJ Open

A scoping review protocol to map evidence on South-South learning exchange in family planning

Journal:	*BMJ Open*
Manuscript ID	bmjopen-2022-061685.R1
Article Type:	Protocol
Date Submitted by the Author:	03-Feb-2023

Complete List of Authors:	Allagh, Komal Preet; World Health Organization, Consultant<br>Kiarie, James; World Health Organization, Department of Reproductive Health and Research<br>Triulzi, Isotta; Scuola Superiore Sant'Anna, Institute of Managment<br>Kabra, Rita ; World Health Organization, Reproductive Health and Research

**Primary Subject Heading**:	Global health
Secondary Subject Heading:	Evidence based practice
Keywords:	Reproductive medicine < GYNAECOLOGY, Public health < INFECTIOUS DISEASES, Protocols & guidelines < HEALTH SERVICES ADMINISTRATION & MANAGEMENT

SCHOLARONE™ Manuscripts

Title: A scoping review protocol to map evidence on South-South learning exchange in family planning

Authors: Komal Preet Allagh[1], James Kiarie[1], Isotta Triulzi[1], Rita Kabra[1]

Affiliations: [1]Department of Reproductive Health and Research including UNDP/UNFPA/UNICEF/WHO/World Bank Special Programme of Research, Development and Research Training in Human Reproduction, World Health Organization, Avenue Appia 20, 1211 Geneva, Switzerland.

Corresponding author: Rita Kabra

Email: kabrar@who.int

For peer review only

For peer review only - http://bmjopen.bmj.com/site/about/guidelines.xhtml

Abstract

Introduction

South-South learning exchange (SSLE) is an interactive learning process where stakeholder teams exchange knowledge and experience to help one, or both to work towards change, by identifying, adopting and/or strengthening implementation of a best practice. SSLE has been conducted between countries to share knowledge on best practices and policies in family planning. To the best of our knowledge, no scoping review has been conducted to synthesize evidence on South-South learning exchange in family planning. In this paper, we outline the protocol to conduct scoping review on South-South learning exchange in family planning.

Methods and analysis

Arksey and O'Malley's scoping review framework with adaptions from Levac et al will be used to guide this scoping review. We will search electronic databases (Medline, Embase, CINAHL, Hinari, ProQuest DB, PUBMED, Web of Science, and WorldCat), grey literature sources and reference lists of included studies. We will focus on literature published till August 2022. The abstract and title screening, full-text screening and data charting will be conducted by two independent reviewers. The findings will be summarized into a narrative based on thematic analysis. Stakeholder interviews will be conducted to understand their perception and experiences in applying South-South learning exchange in family planning.

Ethics and dissemination

The ethics review committee at World Health Organization, Geneva, has exempted this study from ethical approval (ERC.0003752). The findings from the study will provide useful insights into effective approaches, barriers, facilitators to conduct

For peer review only

South-South Learning exchange in family planning. This knowledge will be of significant public health relevance and will help in designing future learning exchanges between countries in the south to accelerate access to quality family planning services. The findings will be disseminated via peer-reviewed journals, conference proceedings, newsletters and workshops.

Keywords: Scoping review, south-south learning exchange, family planning, contraception

Strengths and limitations of this study

- This will be the first scoping review to identify effective approaches, barriers, facilitators to conduct South-South Learning exchange in family planning.
- Stakeholders will be consulted and engaged throughout the review process.
- A comprehensive search strategy is developed with the consultation of a chief librarian to promote a sensitive search.
- The quality of the included studies will not be assessed.

For peer review only

Background

The World Health Organization (WHO) describes South-South learning exchange (SSLE) as an interactive learning process where stakeholder teams exchange knowledge and experience to help one or both teams to work towards change by identifying, adopting and/or strengthening implementation of a best practice [1]. Learning exchange can take place between two teams at the country level or within countries, between provinces, regions, states, districts or local administrative units. The SSLE can offer value to both teams by supporting bi-directional sharing of knowledge, good practices, supporting scaling-up of good practices and capacity building of participants to advocate for the change process. SSLE is often far more convincing and contextually appropriate than learning from publications or experts [2]. Learning exchanges have focused on a wide range of topics in the past, from trade, finance, food security, nutrition and health [3].

Since 2019, WHO has been conducting SSLE in family planning under the WHO Family Planning Accelerator project [4] using a standardized 5-step methodology [1] designed to ensure that SSLE is country-driven, focused on outputs/outcomes and involves rigorous monitoring of the process. The WHO FP Accelerator project builds on the WHO FP Umbrella project that supported over 50 countries to update their national FP guidelines to the latest WHO recommendations [5].

At the International Conference on Population and Development (ICPD), Cairo in 1994, one of the recommendations was that more attention should be given to South-South cooperation as an important instrument of development [6]. Since then, several countries have reported using SSLE referred to as South-South cooperation (SSC), to improve family planning outcomes using different approaches like study tours, site visits, training or expert visits. Together with political dialogue, technical and financial cooperation, SSLE has assisted

For peer review only - http://bmjopen.bmj.com/site/about/guidelines.xhtml

in several knowledge and expertise exchanges through programs, projects and initiatives that have helped solve specific challenges. However, to date, no document highlights and summarizes the key features of an SSLE in family planning, such as the most effective approach to achieve the FP outcomes, the barriers and facilitators to expect from the learning exchange and how to overcome these, and the objectives of the SSLEs that have already taken place.

To the best of our knowledge, there is no existing published synthesis on South-South learning exchange in family planning that incorporates both the reviewed and non-peer-reviewed literature. This scoping review aims to systematically review published and grey literature on SSLE in family planning and identify gaps in the available knowledge to help guide future SSLEs in family planning and research in the field. The study objectives are to review published and grey literature on SSLE in family planning to identify the (i) purposes, (ii) approaches, (iii) key outcomes, (iv) enablers and barriers.

Methods

This scoping review is based on the framework proposed by Arksey and O'Malley's [7] which has been further developed by Levac et al [8]. The framework has six steps: (1) Identification of the research question, (ii)Identification of relevant studies, (iii)Study selection, (iv) Data charting, (v) Collating, summarizing and reporting the results and (vi) Stakeholder consultation

Stage 1: Identification of the research question

This scoping review seeks to answer the following specific research questions:

i. For what purposes have South-South learning exchanges in family planning been used?

For peer review only - http://bmjopen.bmj.com/site/about/guidelines.xhtml

ii. What approaches and methods are used in conducting South-South learning exchange in family planning between countries?

iii. What are the barriers and facilitators encountered in conducting South-South learning exchange in family planning?

iv. What outcomes have been achieved by South-South learning exchange in family planning?

Stage 2: Identification of relevant studies (Search strategy)

With support from the chief librarian at WHO Geneva, the authors will conduct a systematic search within the following electronic databases: Medline, Embase, CINAHL, Hinari, ProQuest DB, PUBMED, Web of Science, and WorldCat. This study will use a complete search strategy (supplementary file 1) that employs keywords, medical subject headings (MeSH) or subject headings search terms that relate to key review concepts: South-South learning exchange, South-South knowledge exchange, South-South exchange, Peer to peer learning exchange, South-South cooperation, information sharing, information exchange, knowledge sharing, knowledge exchange, learning exchange, Family planning, contraception, reproductive health, as well as Boolean terms "AND" and "OR".

No language restrictions will be imposed. Reference lists of the articles will be used to identify more studies using a snowball approach. We will also conduct a grey literature search using Google Scholar and relevant websites (FP 2030, Partners in population and development, UNFPA, WHO, UNOSCC, S-S Galaxy, World Bank and USAID). Types of grey literature that will be retrieved and used in the review include reports, articles, conference proceedings, PhD and Master's thesis, and case studies.

Stage 3: Study Selection

For peer review only - http://bmjopen.bmj.com/site/about/guidelines.xhtml

This review will include studies that meet the defined eligibility criteria (see table 1), published till August 2022. No limits will be placed on study period, language and location of study.

Table 1: Eligibility criteria for the scoping review

Criteria	Inclusion	Exclusion
**Country**	Any country	None
**Date**	Any year	None
**Language**	All languages	None
**Research focus**	Studies that mention objectives, purpose, approaches, process, enablers, barriers, and outcomes of SSLE in family planning.	SSLE on topics other than FP
**Geographical location**	SSLE between countries	SSLE within a country (between institutions, cities & districts)
**Document type**	Scientific report, case study, commentaries, research article, conference proceedings, student thesis, letter to editors and reviews.	Newspaper, power point presentations and magazine articles

Selection process:

We will use a two-part study selection process. First, two independent reviewers (RK and KPA) will screen all titles and abstracts as per inclusion/exclusion criteria. Any article that is considered relevant by either or both reviewers will be included for full-text review. The reviewers (RK and KPA) will then independently assess the full text to determine if the study is included. A third reviewer (JK) will resolve any discrepancies that arise between the two reviewers. Zotero reference manager version 5.0.96.3 [9], a citation and bibliographic software will be used to store and organize all references. The PRISMA flow diagram [10] will be used to report the results of the screening.

Stage 4: Data charting

A data charting form will be prepared in Microsoft Excel to extract study characteristics. KPA and RK will extract all relevant data from the included studies after a thorough reading of the

For peer review only - http://bmjopen.bmj.com/site/about/guidelines.xhtml

full texts. The data charting form will include 14 categories as listed in table 2. This list will be updated when we start reviewing the studies to capture all relevant data to answer the review questions.

Table 2: Data extraction framework

Main category	Description
1. Authors	Name of the authors
2. Title	Title of study
3. Source of publication	Title of the journal or name of publishing organization
4. Year of publication	Year of publication
5. Purpose/objective of SSLE	Describe the stated purpose of the SSLE
6. Year and duration of the SSLE	Indicate the year of SSLE and duration the SSLE lasted
7. Countries participating in the SSLE	List the mentor and mentee countries.
8. Approach used for SSLE	Describe the method used to conduct the learning exchange, example: study tour, virtual, reciprocal exchange, etc.
9. Process of SSLE	Describe the stated process of the SSLE for instance: was a standard method used, planning phase, were stakeholders involved, was an action plan developed, was it implemented, was a follow up done after SSLE was completed.
10. Key stakeholders	Indicate the key FP stakeholders involved in the SSLE in both the countries
11. Reported outputs	Describe the family planning outputs reported in the SSLE (e.g.: capacity building, policy change, etc.)
12. Reported outcomes	Describe the family planning outcomes reported in the SSLE (e.g.: contraceptive prevalence rate, unmet need)
13. Barriers	Describe the factors that inhibit the implementation of the SSLE
14. Facilitators	Describe the factors that supported or enabled the implementation of the SSLE

The data charting form will be pilot tested in a few studies to ensure it is capturing all the information accurately.

Stage 5: Collating, summarizing and reporting the results

A narrative account of the included studies will be prepared to present the literature on SSLE in family planning based on a thematic analysis. Each author will independently review the extracted information to summarize the findings presented across the articles. Relevant themes and sub-themes relating to the study objectives will be developed around the

For peer review only - http://bmjopen.bmj.com/site/about/guidelines.xhtml

following: (i) Purpose of SSLE in family planning, (ii) methods and process used for SSLE in family planning, (iii) FP outputs and outcomes achieved from SSLE in family planning and (iv) Barriers and enablers of SSLE in family planning.

Stage 6: Stakeholder Consultation

Levac et al. suggested that the consultation stage provides opportunities for stakeholder involvement, providing insights beyond what is reported in the literature [6]. The consultations will aim to understand stakeholder perception and experiences in applying SSLE in family planning. Following ethical approval, one reviewer (IT) will identify and invite the authors of the included studies for the interviews. In addition, a snowball approach will be used to identify more experts. We will interview 12-20 individuals. All interviews will be conducted virtually via google meet for an approximate duration of 45-60 minutes and will be recorded using a voice recorder and through taking notes. Participants will be assured of confidentiality and verbal informed consent will be recorded. Each interview will be transcribed verbatim. Relevant themes and sub-themes relating to the study objectives will be identified using RQDA software for qualitative analysis [11].

The interviewer will provide an overview of the scoping review. A semi-structured interview guide is developed to understand the experiences and views of stakeholders on SSLE in family planning. The questionnaire includes a set of open-ended questions (supplementary file 2) to guide the discussion and will cover the following topics: (i) role of the organization in SSLE and the process used (ii) purpose of SSLE and approaches used (iii) views and experiences on SSLE in FP (iv) perception on challenges and successes observed during the SSLE process and (v) lessons learnt.

Patient and public involvement

No patients are involved. The study is a review of literature, there are no study participants.

For peer review only - http://bmjopen.bmj.com/site/about/guidelines.xhtml

Discussion

The proposed review aims to map evidence on South-South learning exchange in family planning. The extracted data will be presented in a narrative and tabular form to cover all the review objectives. The results of this scoping review will build evidence on effective approaches, barriers, facilitators and key outcomes achieved in SSLE in family planning and may reveal further research areas. Results of the scoping review will be disseminated in a peer-reviewed journal and other ways such as through consultations, newsletters, conferences and workshops.

Ethics and dissemination

The ethics review committee at World Health Organization, Geneva, has exempted this study from ethical approval (ERC.0003752). The findings from the study will provide useful insights into effective approaches, barriers, facilitators to conduct South-South Learning exchange in family planning. This knowledge will be of significant public health relevance and will help in designing future learning exchanges between countries in the south to accelerate access to quality family planning services. The findings will be disseminated via peer-reviewed journals, conference proceedings, newsletters and workshops.

For peer review only

For peer review only - http://bmjopen.bmj.com/site/about/guidelines.xhtml

Abbreviations

FP- Family planning

PRISMA- Preferred Reporting Items for Systematic Reviews and Meta-Analyses

PPD- Partners in Population and Development

SSLE- South-South learning exchange

SSC- South-South Cooperation

S-S Galaxy- South-South Galaxy

WHO- World Health Organization

UNFPA- United Nations Population Fund

UNOSCC- United Nations Office of South-South Cooperation

USAID- United Nations Agency of International Development

Funding:

This work received funding from the World Health Organization (WHO) through the WHO FP Accelerator Project 2019-2022 supported by the Bill and Melinda Gates Foundation [OPP1203035].

The content is solely the responsibility of the authors and does not necessarily represent the official views of the funders. Any opinion, finding, conclusion or recommendation expressed in this material is that of the authors.

Conflict of Interest:

The authors declare no conflict of interest.

Contributions:

KPA prepared the first draft with significant contributions from JK, IT and RK. All authors reviewed the draft manuscript and approved the final manuscript for publication.

For peer review only - http://bmjopen.bmj.com/site/about/guidelines.xhtml

References:

1. IBP Network and World Health Organization. Family planning Accelerator project: A Step-by-Step Guide to South-South learning exchanges. Geneva. World Health Organization. Retrieved on 27th January 2022 from https://ibpnetwork.org/media_center/folder/47ae9fb7-bab0-41fd-8a7d-fb8fd7d65018
2. The World Bank. The South-South experience exchange facility: Implementation progress report. 2018. Retrieved on 1 February 2023 from https://documents1.worldbank.org/curated/en/744941570117614575/pdf/The-South-South-Experience-Exchange-Facility-Implementation-Progress-Report-2018.pdf
3. EnCompass. South-South cooperation: Expanding opportunities for innovative solutions- Issue brief. November 2010. Retrieved on 4 Nov 2021 from https://encompassworld.com/wp-content/uploads/2016/04/southtosouthissuebrief.pdf
4. World Health Organization. WHO Family planning Accelerator project. February 2020. Retrieved on 22nd March 2021 from https://www.who.int/reproductivehealth/projects/WHO-FP-Accelerator-Project-Brief.pdf?ua=1
5. Kabra R., Ali M., Kiarie J. Design and initial implementation of the WHO FP umbrella project- to strengthen contraceptive services in the sub-Saharan Africa. Reproductive Health. 2017. 14:74, DOI 10.1186/s12978-017-0335-0. Accessed on 24 December 2021 from https://reproductive-health-journal.biomedcentral.com/track/pdf/10.1186/s12978-017-0335-0.pdf

For peer review only

6. Evans I. What do you do, Partner? The Lancet. 1996, 348: 211-212

7. Arksey H, O'Malley L. Scoping studies: towards a methodological framework. International Journal of Social Research Methodology. 2005; 8:19-42.

8. Levac D, Colquhoun H, O'Brien KK. Scoping studies: advancing the methodology. Implementation Science 2010; 5: 69.

9. Zotero. Retrieved on 27th January 2022 from https://www.zotero.org/

10. PRISMA-Transparent reporting of Systematic reviews and meta-analyses. Retrieved on 27th January 2022 from http://prisma-statement.org/prismastatement/flowdiagram.aspx

11. What is RQDA and what are its features? Retrieved on 27th January 2022 from http://rqda.r-forge.r-project.org/

For peer review only

**1. Database: PubMed**<br>Database Provider: http://www.pubmed.gov<br>Date limits: none<br>Language limits: none<br>Other notes on the search:
("South-South" OR ("International Cooperation"[MH] AND "Developing Countries"[MH])) AND ("learning exchange" OR " knowledge exchange" OR "Peer to peer " OR "cooperation" OR "information sharing" OR "information exchange" OR "knowledge sharing" OR "knowledge exchange" OR "learning exchange") AND (reproduct* OR "family planning" OR contracept* OR "Family Planning Services" OR "Reproductive Medicine" OR "Population Control" OR "Population Growth" OR Contraception OR Fertility OR "Contraception Behavior" OR "Embryo Transfer" OR "Intrauterine Devices" OR "Long Acting Reversible Contraception" OR "Maternal Child Health Centers" OR "Pregnancy In Adolescence" OR "Reproductive Techniques Assisted")
**2. Database: EMBASE**<br>Database Provider: http://www.embase.com<br>Date limits: none<br>Language limits: none<br>Other notes on the search:
('South-South' OR ('developing country'/exp AND 'international cooperation'/exp/mj)) AND ('learning exchange' OR ' knowledge exchange' OR 'Peer to peer ' OR 'cooperation' OR 'information sharing' OR 'information exchange' OR 'knowledge sharing' OR 'knowledge exchange' OR 'learning exchange') AND (reproduct* OR 'family planning' OR contracept* OR 'Family Planning Services' OR 'Reproductive Medicine' OR 'Population Control' OR 'Population Growth' OR Contraception OR Fertility OR 'Contraception Behavior' OR 'Embryo Transfer' OR 'Intrauterine Devices' OR 'Long Acting Reversible Contraception' OR 'Maternal Child Health Centers' OR 'Pregnancy In Adolescence' OR 'Reproductive Techniques Assisted')

For peer review only

**3. Database: Global Index Medicus (GIM)**<br>Database Provider: http://www.globalhealthlibrary.net/<br>Date limits: none<br>Language limits: none<br>Other notes on the search: Filter activated to limit to Regional Databases (AIM, LILACS, IMEMR, IMSEAR & WPRIM)
("South-South" OR south*south OR south?south OR (mh:("International Cooperation") AND mh:("Developing Countries"))) AND (reproduct* OR "family planning" OR contracept* OR "Family Planning Services" OR "Reproductive Medicine" OR "Population Control" OR "Population Growth" OR Contraception OR Fertility OR "Contraception Behavior" OR "Embryo Transfer" OR "Intrauterine Devices" OR "Long Acting Reversible Contraception" OR "Maternal Child Health Centers" OR "Pregnancy In Adolescence" OR "Reproductive Techniques Assisted")
**4. Database: CINAHL**<br>Database Provider:http://www.ebsco.com<br>Date limits: none<br>Language limits: none<br>Other notes on the search:
("South-South" OR ("International Cooperation" AND "Developing Countries")) AND (reproduct* OR "family planning" OR contracept* OR "Family Planning Services" OR "Reproductive Medicine" OR "Population Control" OR "Population Growth" OR Contraception OR Fertility OR "Contraception Behavior" OR "Embryo Transfer" OR "Intrauterine Devices" OR "Long Acting Reversible Contraception" OR "Maternal Child Health Centers" OR "Pregnancy In Adolescence" OR "Reproductive Techniques Assisted")

**5. Database: HINARI**<br>Database Provider: https://www.who.int/hinari/en/<br>Date limits: none<br>Language limits: none

For peer review only

Other notes on the search: Search beyond your collection<br>Limit excluded newspaper articles
(TitleCombined:("south to south" OR "south south")) AND (("family planning" OR contracept* OR "Reproductive Medicine" OR "Population Control" OR "Population Growth" OR Contraception OR "Maternal Child Health Centers" OR "Pregnancy In Adolescence" OR "birth control"))
**6. Database: Web of Science**<br>Database Provider: https://www.webofscience.com/wos/woscc/<br>Date limits: none<br>Language limits: none<br>Other notes on the search:
(ALL="South-South" OR (TS="International Cooperation" AND TS="Developing Countries")) AND ALL=(reproduct* OR "family planning" OR contracept* OR "Family Planning Services" OR "Reproductive Medicine" OR "Population Control" OR "Population Growth" OR Contraception OR Fertility OR "Contraception Behavior" OR "Embryo Transfer" OR "Intrauterine Devices" OR "Long Acting Reversible Contraception" OR "Maternal Child Health Centers" OR "Pregnancy In Adolescence" OR "Reproductive Techniques Assisted")
**7. Database: WorldCat**<br>Database Provider: https://www.worldcat.org/<br>Date limits: none<br>Language limits: none<br>Other notes on the search: Non-journal articles
'ti:("South-South" OR ("International Cooperation" AND "Developing Countries")) AND (reproduct* OR "family planning" OR contracept* OR "Family Planning Services" OR "Reproductive Medicine" OR "Population Control" OR "Population Growth" OR Contraception OR Fertility OR "Contraception Behavior" OR "Embryo Transfer" OR "Intrauterine Devices" OR "Long Acting Reversible Contraception" OR "Maternal Child Health Centers" OR "Pregnancy In Adolescence" OR "Reproductive Techniques Assisted")'

For peer review only

Interview guide for Stakeholder consultations on SSLE in family planning

May I remind you that:

1) The interview will be audio recorded to ensure all information is captured. I would like to ask for your consent before we proceed.
2) You are free to refuse to participate and may withdraw your consent or discontinue participation at any time. The refusal to participate will not result in any penalty or loss of benefits. You can also skip any questions you do not want to answer.

Name of Participant:

Organization and designation:

Date & time of interview:

For peer review only

<u>Ice breaker</u>

1. Welcome and introductions
2. Describe the role of your organization in the South-South Learning Exchange.
3. Describe your role in the SSLE process and the number of SSLEs you were involved in.

<u>Purpose of SSLE in family planning</u>

4. Explain the main goal of each SSLE process you were involved in.

   Probes: Area: health system service, health commodities, human resource management and development, health care delivery, health care financing, health information system, etc

<u>Process of SSLE in family planning</u>

5. How did the process begin?

   Probes: How were the countries selected? By whom? Who were the knowledge seekers and providers?
6. Why did the process begin?

   Probes: How did the country identify the learning area/ objective of the SSLE? Did the country conduct a needs assessment/situational analysis?
7. When did this SSLE take place, and how long was the duration?
8. List the stakeholders involved in SSLE and their roles.
9. Who were the main champions of the process (NGO, Ministry of Health, etc.)?
10. Did you follow a standardized process for conducting the SSLE?

For peer review only - http://bmjopen.bmj.com/site/about/guidelines.xhtml

Probes: Was a structured approach or a Standard Operation Procedure used to guide the SSLE? Describe the approach adopted throughout the process.

11. Describe how the process was monitored and how were the results measured?

    Probes: how were the indicators selected and tracked, and how often?

<u>Key outcomes</u>

12. What were the family planning outcomes achieved from SSLE?

    Probes: Were policies, guidelines, capacity building or quality of services achieved at the end of the SSLE process? Was the planned goal achieved?

<u>Barriers and enablers</u>

13. In your opinion, what were the barriers faced during the SSLE process?
14. What were the facilitators that helped during the SSLE process?

<u>Final question</u>

15. Describe the main lessons learnt from your experience with SSLE?

For peer review only

For peer review only - http://bmjopen.bmj.com/site/about/guidelines.xhtml
